# Identification and validation of novel signature associated with hepatocellular carcinoma prognosis using Single-cell and WGCNA analysis

**DOI:** 10.7150/ijms.79274

**Published:** 2023-05-11

**Authors:** Hang Song, Yang Ge, Jing Xu, Rui Shen, Peng-cheng Zhang, Guo-quan Wang, Bin Liu

**Affiliations:** 1 Department of Biochemistry and Molecular Biology, School of Integrated Chinese and Western Medicine, Anhui University of Chinese Medicine, 230012, Hefei, China.; 2 Department of oncology, The Third Affiliated Hospital of Zhejiang Chinese Medical University, 219 Moganshan Road, Xihu District, Hangzhou City, Zhejiang Province 310005, China.; 3 Cancer Research Centre, Beijing Chest Hospital, Capital Medical University/Beijing Tuberculosis and Thoracic Tumor Research Institute, 101149, Beijing, China.

**Keywords:** HCC, Necroptosis, Prognosis, TCGA, WGCNA

## Abstract

**Background:** Hepatocellular carcinoma is a rapidly advancing malignancy with a poor prognosis. Therefore, further research is needed on its potential pathogenesis and therapeutic targets.

**Methods:** In this study, the relevant datasets were downloaded from the TCGA database and the key modules were identified using WGCNA in the necroptosis-related gene set, while single-cell datasets were scored using the necroptosis gene set. Differential genes in the high- and low-expression groups were determined using the WGCNA module genes as intersection sets to identify key genes involved in necroptosis in liver cancer. Then, prognostic models were constructed using LASSO COX regression followed by multi-faceted validation. Finally, model genes were found to be correlated with key proteins of the necroptosis pathway and used to identify the most relevant genes, followed by their experimental validation. Subsequently, on the basis of the analysis results, the most relevant SFPQ was selected for cell-level verification.

**Results:** We constructed a prognosis model that included five necroptosis-related genes (EHD1, RAC1, SFPQ, DAB2 and PABPC4) to predict the prognosis and survival of HCC patients. The results showed that the prognosis was more unfavorable in the high-risk group compared to the low-risk group, which was corroborated using ROC curves and risk factor plots. In addition, we further checked the differential genes using GO and KEGG analyses and found that they were predominantly enriched in the neuroactive ligand-receptor interaction pathway. The results of the GSVA analysis demonstrated that the high-risk group was mainly enriched in DNA replication, regulation of the mitotic cycle, and regulation of various cancer pathways, while the low-risk group was predominantly enriched in the metabolism of drugs and xenobiotics using cytochrome P450. SFPQ was found to be the main gene that affects the prognosis and SFPQ expression was positively correlated with the expression of RIPK1, RIPK3 and MLKL. Furthermore, the suppression of SFPQ could inhibit hyper-malignant phenotype HCC cells, while the WB results showed that inhibition of SFPQ expression also resulted in lower expression of necroptosis proteins, compared to the sh-NC group.

**Conclusions:** Our prognostic model could accurately predict the prognosis of patients with HCC to further identify novel molecular candidates and interventions that can be used as alternative methods of treatment for HCC.

## Introduction

Hepatocellular carcinoma (HCC) is among the deadliest types of cancer worldwide and was the fastest-growing malignancy until a few years ago. Recently, the rates of morbidity and mortality of HCC have been on the decline, with 2022 data showing that the incidence of HCC had declined by approximately 2% per year from 2014 to 2018, although the overall number of new cases and deaths remain high^1^. Treatment for HCC is currently based on therapy, such as sorafenib, levatinib, and regorafenib; surgical treatment methods, such as hepatectomy, liver cancer ablation, and liver transplantation; radiotherapy; as well as other types of treatment, such as percutaneous ethanol injections; and liver preservation therapy^2^-^4^. However, the prognosis for HCC remains bleak. Many studies have confirmed some of the mechanisms that underlie the development of HCC, but more breakthroughs are still needed to enrich patient clinical care. In this regard, there has been a challenge in determining the heterogeneity and complexity of HCC. Therefore, there is a need to identify specific expression modules in HCC to help improve the treatment and prognosis of patients with HCC.

Apoptosis, autophagy, and necroptosis are the three most studied manifestations of programmed cell death, with Chan et al. first introducing the concept of "necroptosis" in 2003^5^. Receptor-interacting serine/threonine-protein kinase 1 (RIPK1) is a key mediator of cell death and inflammation. Interact with Receptor Interacting Serine/Threonine Kinase 3 (RIPK3), their kinase-mediated necrosis is referred to as necroptosis. In necroptosis process, mixed lineage kinase domain-like protein (MLKL) also emerges as executioner in it, plays in a RIPK3-dependent form of regulated necrosis. So, necroptosis is mediated by the RIPK1/RIPK3/MLKL axis and has the morphological characteristics of a necrotic cell. It was found that, similar to apoptosis, necroptosis is tightly regulated by intracellular signaling factors, of which RIPK1 and RIPK3 are key regulators. The combination of these two factors recruits MLKL and triggers a conformational change, which ultimately lead to cell rupture^6^, ^7^. Therefore, necroptosis studies are often evaluated by detecting RIP1, RIP3, and their binding. Recent studies have shown that necroptosis is associated with the pathogenesis of several diseases. In response, inhibitors of necroptosis, such as RIPK1 inhibitors (Nec-1)^8^, RIPK3 inhibitors (9GSK'840)^9^, and MLKL inhibitors (Necrosulfonamide)^10^, have been developed and offer an opportunity for the discovery of novel molecular biomarkers and treatment targets. Furthermore, necroptosis participates in tumour metastasis and regulates tumour growth^11^, ^12^. Seifert et al.^13^ reported that necroptosis can contribute to tumor progression and that as key pathway proteins of necroptosis, RIPK1 and RIPK3, are essential for tumor development. MLKL mediates enzyme activation and promotes necroptosis^14^. Studies have also shown that pancreatic ductal adenocarcinoma cells promote tumor growth and proliferation by creating an immunosuppressive microenvironment through necroptosis^15^. Up-regulation of RIPK3 expression can inhibit the immunosuppressive activity of tumor-associated macrophages to a certain extent, which can suppress HCC tumorigenesis^16^. The inflammatory response triggered by necroptosis can increase the development of fibrosis in mouse models and promote the development of HCC^17^. Furthermore, the findings of Li et al. and Chen et al. were in agreement with the results of the above-mentioned studies^18^, ^19^. Therefore, we focus on necroptosis as a potential predictor of tumor prognosis.

In this study, we obtained information on HCC patients from the GEO and TCGA databases. A prognostic model of necroptosis in HCC was constructed, and five genes were identified that were significantly associated with the prognosis. Furthermore, to further clarify the effect of the model on tumor prognosis, SFPQ was identified as the main gene that affects prognosis (HR = 1.828, p < 0.004). Next, to verify the correlation between SFPQ and necroptosis, SFPQ was found to be directly positively correlated with RIPK1 (r = 0.499, p < 0.001), RIPK3 (r = 0.313, p < 0.001), and MLKL (r = 0.427, p < 0.001), suggesting that SFPQ may be a driver gene that promotes necroptosis. To further determine the effect of SFPQ in HCC, we engineered corresponding shRNAs that could target the SFPQ gene, and the result showed that inhibition of SFPQ expression could inhibit the malignant phenotype of HCC cells, while the WB results demonstrated a relatively lower protein expression in the sh-SFPQ group, compared to the sh-NC group. These outcomes may allow the identification of novel legal targets and intervention strategies for the management of HCC.

## Materials and Methods

### Single cell data acquisition and processing

The single cell GSE149614 dataset was downloaded from the GEO database and included data on samples obtained from 10 patients with primary liver cancer tumors, 2 patients with portal vein cancer thrombosis, 1 patient with metastatic lymph nodes and 8 patients with normal liver tissue. Raw data contained a total of 25,479 genes and 71,915 cells. The percentages of mitochondria, ribosomes and erythrocytes were calculated using the PercentageFeatureSet function with > 300 genes expressed per cell. Mitochondrial gene expression was less than 15%, ribosomal gene percentage was > 3%, while erythrocyte gene percentage was less than 1%. The total number of cells after filtering was 69,997 Supplementary Figures 1-2). The merged ScRNA-seq data were first normalized and the top 2000 highly variable genes were identified using the FindVariableFeature function, while all genes were scaled using the ScaleData function, and the top 2000 highly variable genes were filtered and downscaled using the RunPCA function. Then batch correction was performed using the harmony algorithm. Cells were clustered using the "FindNeighbors" and "FindCluster" functions (resolution = 0.8) to identify clusters of cells (Supplementary figure 3. Next, we used the UMAP method for further reduction in dimensionality. Finally, we selected 24 subgroups of marker genes using the FindAllMarkers function, where Minpct = 0.25 (the expression ratio of the least differential gene). Finally, the marker genes were selected using a corrected p < 0.05.

### Transcriptome data acquisition and processing

TCGA data were downloaded to be used as the training cohort using the "TCGAbiolinks" package^20^, while the LIHC data type was counts. A total of 424 transcriptomic data samples with complete clinical data were used. The LIHC dataset, GSE14520, was downloaded to be used as the validation set from the GEO database and used for the external validation of the model.

### Acquisition of necroptosis genes

A total of 626 necroptosis-related genes were obtained from the Genecards database^21^ and a total of 99 genes were found to have a correlation score greater than 11.

### Single cell data necroptosis correlation score

The AddModuleScore^22^ function was used to calculate the average expression value of necroptosis genes in each cell of the single cell data, and the single cell data were classified into high and low expression groups according to the median expression value.

### Weighted gene co-expression network analysis (WGCNA)

WGCNA^23^, ^24^ is a systems biology analysis method used to characterize gene association patterns between different samples and can be used to identify highly synergistic sets of genes and candidate biomarker genes or therapeutic targets based on the endogeneity of the gene set and the association between the gene set and the phenotype. In this experiment, the ssGSEA^25^ algorithm was used to assess the scoring of necroptosis genes in the LIHC expression matrix, and WCGNA was used to identify modules that are closely associated with necroptosis and to determine modular genes^26^-^28^.

### Construction of a prognostic model of necroptosis

Minimizing absolute contraction and the regression of the selection operator LASSO^29^ regression are machine learning algorithms that are commonly used at present to construct diagnostic models, using regularization to determine the occurrence of overfitting during curve fitting and to improve the accuracy of the model. Based on the necroptosis genes selected, we used one-way Cox regression analysis to screen genes with prognostic significance, followed by LASSO regression analysis (glmnet package^30^) to select the genes that were ultimately used to construct the prognostic model. The model equation was as follows. Risk score (patient) = Σi Coefficient (gene) × Expression (gene). The prognostic genes were classified into high and low expression groups based on the median expression value and the survival of each gene.

### Prognostic model assessment

The prognostic genes were found to be expressed in the high and low risk groups, based on the median value of the risk score, which was used to categorize the high and low risk groups. Risk factors were plotted using the pheatmap package, while survival curves were plotted for the high and low risk groups using the survminer package, and AUC areas at 1, 3, and 5 years were calculated using the time ROC package off R software^31^. The predictive power of the model was validated using the GSE14520 dataset.

### Nomogram construction

A nomogram was constructed to assess the risk of death in patients with LIHCC by combining clinical data with model risk score values. The results were then verified using a review of the prognostic ROC curve.

### GO/KEGG/GSVA analysis of the high- and low-risk groups

Based on the grouping information of the high and low risk groups, differential analysis was performed using the Deseq2 R package^32^ to screen for differential genes with a p-value of less than 0.05 and an absolute logFC value greater than 1. Using the clusterProfiler R package^33^ for Gene Ontology (GO) annotation analysis of gene ontology (GO) and the Kyoto Encyclopedia of Genes and Genomes (KEGG)^34^ pathway enrichment analysis of differential genes, a critical value of p < 0.05 for FDR was considered to indicate statistical significance. To investigate differences in biological processes between the high and low risk groups, an enrichment analysis was performed using GSVA^35^-^37^ based on a dataset containing the gene expression profiles of LIHC patients. The set of gene "c2.cp.kegg.v7.5.1.symbols.gmt" was downloaded from the MSigDB database for GSVA analysis and a p < 0.05 was considered to indicate significant enrichment.

### Experimental validation

**a) Cell culture** Human-derived HCC cells, MHCC97H, and Huh-7 cell culture were provided by the School of Integrative Medicine of the Anhui University of Chinese Medicine. Cells were cultured in a high glucose DMEM medium (Corning, USA) containing 10% FBS (Gibco, USA) and 1% penicillin-streptomycin (Hycolne, Uruguay) at 37 ° C in a 5% CO_2_ atmosphere.

**b) Transfection** Lentiviral packaging was provided by Biotech (China). A Lipofectamine 2000 system (Invitrogen) was used to transfect sh-NC and sh-SFPQ, according to the manufacturer's instructions.

**c) RT-qPCR** After cell transfection, total RNA was extracted from all cells using a Total RNA Extraction Kit (Solarbio). Then, cDNA was obtained through reverse transcription using the Reverse Transcription Assay Kit (TAKARA), according to the manufacturer's instructions. A qPCR kit (TAKARA) was used to perform the experiments. Expression data were normalized to the expression level of β-actin using the 2^-ΔΔCt^ method.

**d) MTT experiment** The cells were inoculated at a density of 5 × 10^3^ cells/well into 96-well plates and incubated overnight. The cells were then transfected for 12, 24, 48, and 72 h. A total of 10 μL of CCK-8 solution was added to each well and incubated with the cells for 4 h. The absorbance was measured at 450 nm using a spectrophotometer.

**e) EdU experiment** The cells were inoculated at a density of 1 × 10^5^ cells/well into 24-well plates and incubated overnight. Transfection was performed separately for up to 24 h. Then, Edu solution, 4% paraformaldehyde, 2 mg/ml glycine, 0.5% Tritonx-100, staining reaction solution, and DAPI were sequentially added, according to the manufacturer's instructions (Sangon Biotech, China). Finally, cells were washed twice with PBS. After staining was completed, the images were captured under a fluorescence microscope.

**f) Wound healing experiment** Lines were drawn using a marker at the bottom of each well of the 6-well plates for positioning. Cells were spread in the 6-well plate and when their density reached 90%, cells were transfected and scored. The images of the well plates were captured at 0 and 24 h, respectively. Calculation formula: Mobility (%) = (0 h width - 24 h width)/0 h width x 100.

**g) Trans-well experiments** The cells were digested and counted within 24 h after transfection treatment and added to the upper chamber after resuspension with a serum-free medium at a density of 2 x 10^4^ cells/well per chamber. In the lower chamber, 600 μL of the medium containing 10% FBS was added. After 24 h of incubation, 500 μL of methanol was added for 30 min to fix the cells. Crystalline violet was added and kept overnight for staining, and excess was removed. The chambers were allowed to dry, images were captured under a microscope, and cell counts were recorded.

**h) WB experiment** After cell transfection, cellular proteins were extracted using RIPA and PMSF (Beyotime, China), and protein quantification was performed using a BCA quantification kit (Thermofisher,). The experiments were conducted based on the WB basic method approach. The following steps were performed: preparation of PAGE gels, loading and electrophoresis, protein transfer, membrane closure, incubation of primary antibodies, incubation of secondary antibodies, and finally detection using ECL chemiluminescence detection kits (Novozymes Bio). Antibodies against RIPK3, MLKL and β-actin were purchased from Abcam (ab226297, ab243142 and ab115777), while RIPK1 antibodies were purchased from Invitrogen (PA5-29223).

### Statistical analysis

SPSS 17.0 software was used for statistical analysis. All data were replicated from three independent experiments and presented in the form of mean and standard deviation (mean +/- Standard error of the mean) after homogenization. One-way ANOVA was performed for comparisons between multiple groups, followed by an LSD-t test. p < 0.05 was considered to indicate statistical significance.

## Results

### Single-cell data analysis

**Figure 1:**
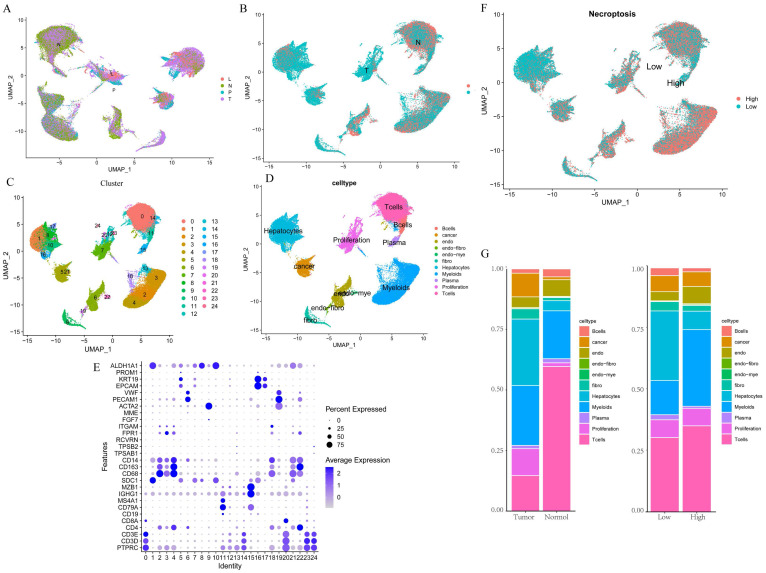
Single-cell data analysis of GSE149614. A. Distribution of samples from four different sources; B. Tumor versus normal distribution; C. Umap of the 24 cell clusters; D. Cell subgroups after labeling by marker genes; E. Marker genes for different subgroups; F. Cell distribution in the high and low necroptosis scoring groups; G. Plot of tumor versus normal and the proportion of cells in the high- and low-necroptosis scoring group.

We first integrated the single-cell dataset, GSE149614, and it showed a good level of integration (Figure 1A) with no significant batch effects. Then, the dataset was divided into two clusters based on tumor vs. normal (Figure 1B), and yielded a total of 2000 differential genes between the two clusters, which were compared. Subsequently, the dataset was divided into 24 clusters (Figure 1C), and by reviewing the literature, the dataset was finally separated into 11 cell types based on the marker gene of the different cells, namely B cells, Cancer cells, Endothelial, Endo- Fibroblasts cells, Endo- Myeloids cells, Fibroblasts, Hepatocytes, Myeloids cells, Plasma cells, Proliferation cells, and T Cells (Figure 1D-E). Then, the cells were further divided into high and low expression groups based on necroptosis gene scores, and the resulting 1,534 differential genes were obtained and compared, followed by screening for genes with p < 0.05, Log2 FC > 1, which yielded a total of 767 differential genes. Subsequent analysis of cell fractions in tumor versus normal and high and low risk groups revealed a prominently lower percentage of T cells in Tumor group and an increase in the percentage of T cells and myeloid cells in patients in the group with high necroptosis score, compared to the group with low score (Figure 1G).

### WGCNA analysis

**Figure 2:**
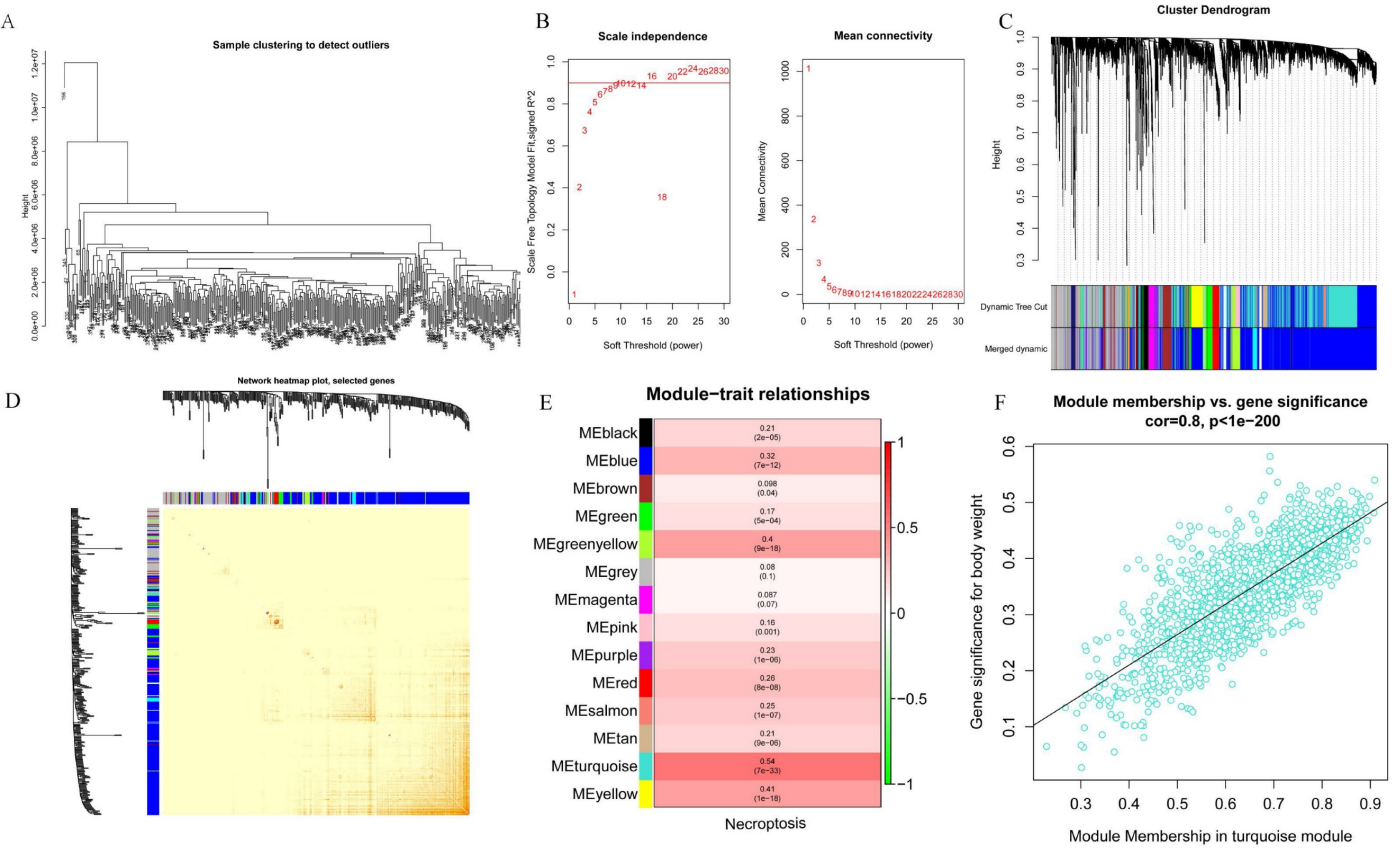
Screening for modules associated with necroptosis based on WGCNA analysis. A. Sample clustering to detect outliers; B. Soft threshold settings; C. The scale-free fit index for soft-thresholding powers. D. Module correlations. E. Each module correlates with module genes; F. MEturquoise module correlates with genes.

WGCNA analysis was performed on the 424 samples obtained from the LIHC cohort of the TCGA database to determine the modules associated with necroptosis. Outliers were removed by sample grouping (Figure 2A), the soft threshold was set at 7 (Figure 2B), the minimum gene expression of the module was set at 30 and mergeCutHeight was set at 0.25. A total of 14 modules were obtained using the hierarchical clustering method with an average linkage (Figure 2C), and the correlation heat map of the modules was constructed (Figure 2D). Subsequently, we used ssGSEA to assess correlations between necroptosis-related modules and found that the MEturquoise, MEyellow, and MEgreenyellow modules were strongly correlated with necroptosis (Figure 2E). Then, the module-gene correlations were calculated and we found that the MEturquoise module was the most strongly correlated (cor=0.8 p=1e-200) (Figure 2F).

### Prognostic model construction

**Figure 3:**
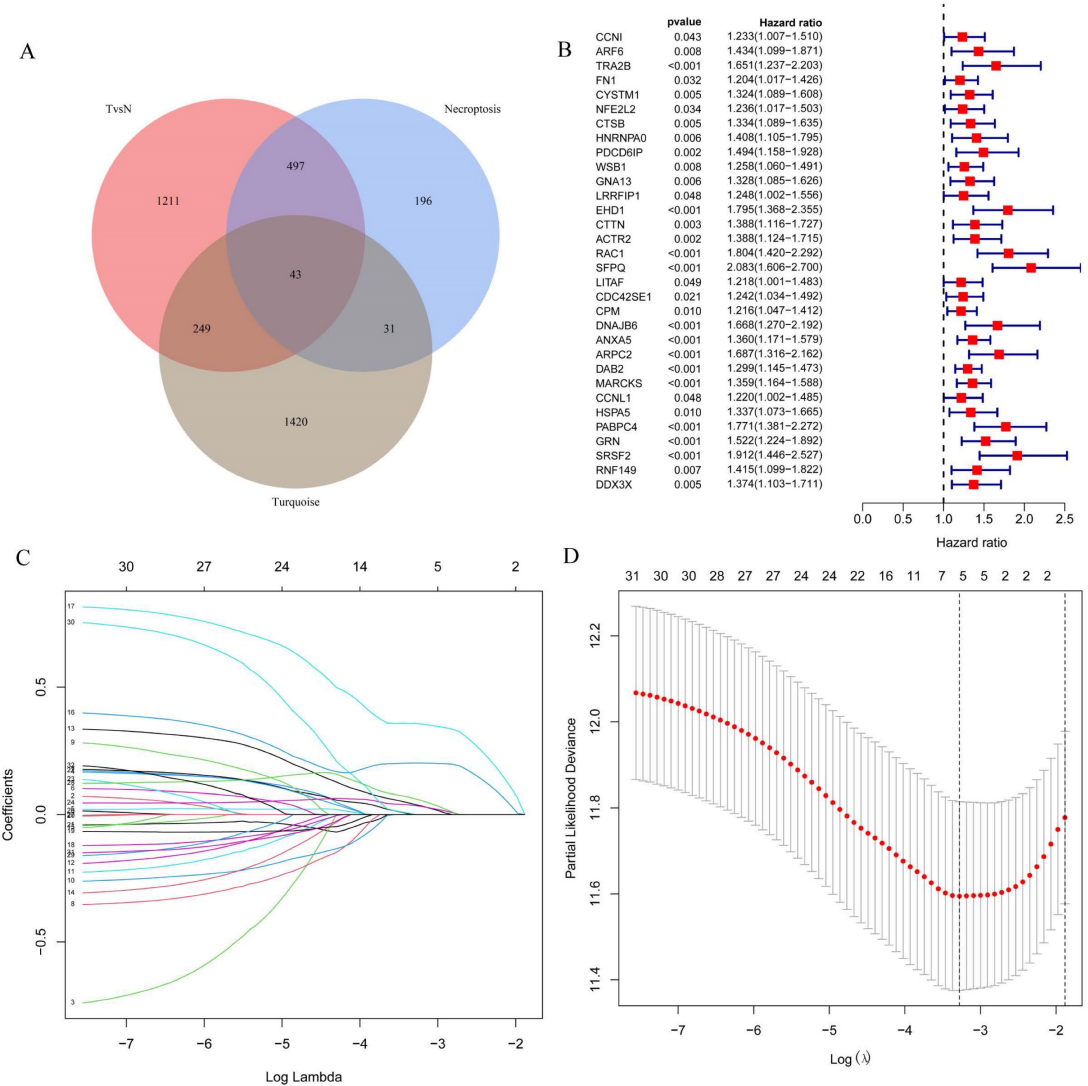
Screening for key genes associated with tumourigenesis to construct prognostic models. A. Venn diagram were obtained for a total of 43 differential genes; B. One-way COX regression analysis to screen for prognostic genes; C. LASSO coefficient profile plots of each gene; D. The partial likelihood deviance for the LASSO Cox regression analysis.

The 43 differential oncogenes associated with necroptosis were targeted by intersecting the MEturquoise module with the differential genes obtained from the single-cell dataset (Figure 3A). To screen for key genes associated with tumorigenesis, we performed the differential expression analysis of gene expression data obtained from the TCGA database using the R package, LIMMA. All 33 differential genes that were significantly associated with prognosis were screened using univariate COX regression (Figure 3B), followed by further gene screening using Lasso COX regression analysis, with the trajectory of each gene shown in the figure (Figure 3C). Meanwhile, a value of one was added to the raw matrix values of the TCGA data and the logarithm of 2 was used as the processed value before analysis. Finally, we identified five genes that were significantly associated with the prognosis: EHD1, RAC1, SFPQ, DAB2 and PABPC4 (Figure 3D) (Table 1). The formula used was risk score = 0.0389*EHD1 + 0.2019*RAC1 + 0.3577*SFPQ + 0.0352*DAB2 + 0.0613*PABPC4.

### Differential expression and cellular localization of the prognostic genes

**Figure 4:**
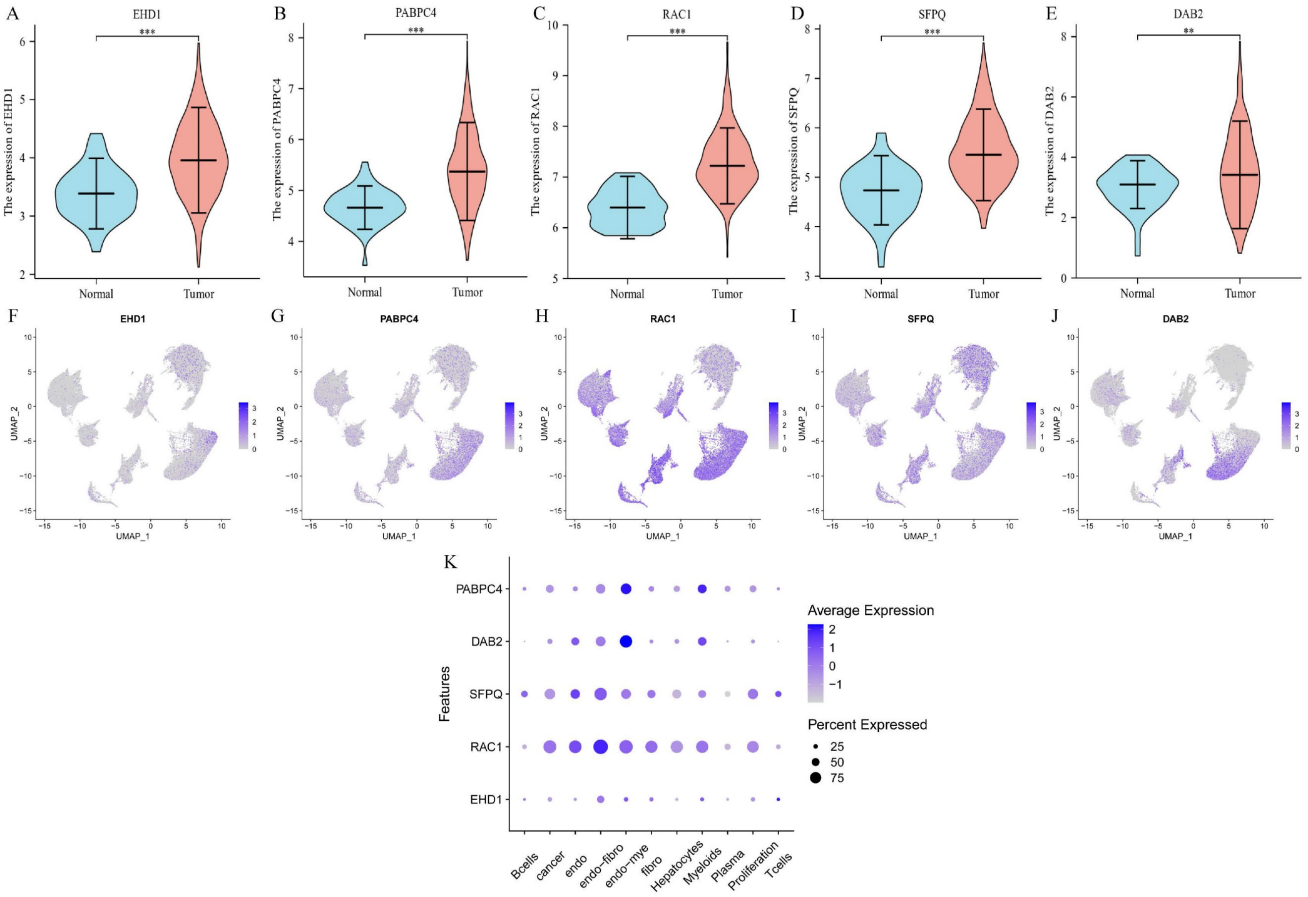
Differential expression and cellular localization of prognostic genes. A-E. Genes were highly expressed in tumors, in order of EHD1, PABPC4, RAC1, SFPQ and DAB2 (p<0.05); F-K. Genes were localized in cells, in order of EHD1, PABPC4, RAC1, SFPQ and DAB2. (*p<0.05, **p<0.01, ***p<0.001).

We determined the expression of each gene separately in tumor tissues and normal tissues using the TCGA database, and the five prognostic genes were found to be highly expressed in tumor tissues (p < 0.01) (Figure 4A-E). Then we explored the expression of each gene in the cells and found that EHD1 was expressed in Endo-fibroblasts cells; PABPC4 in Endo- Myeloids cells and Hepatocytes; RAC1 in endothelial, Endo-fibroblasts cells, and Endo-Myeloids cells; SFPQ in Endo-fibroblasts cells and proliferation cells; and DAB2 in Endo-Fibroblasts cells.

### Prognostic model of survival analysis and clinical correlation analysis

**Figure 5:**
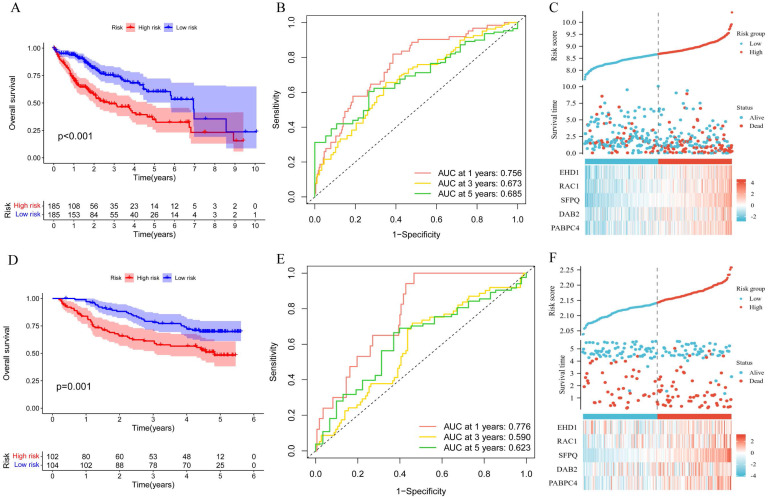
Correlation of prognostic models with clinical characteristics and survival analysis. A. Survival analysis of prognostic models; B.ROC analysis; C. Risk factor plots. D-E. GSE14520 validation set validation.

**Figure 6:**
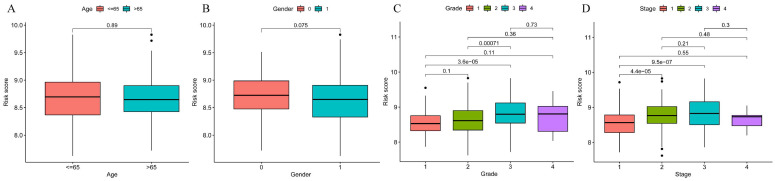
Prognostic model and clinical correlation analysis. A-B. Prognostic model and age ethnicity are not statistically different (p>0.05); C-D. Prognostic model and Grade and stage correlation, the difference is statistically significant (p<0.05).

To explore the significance of the model in guiding the patient's prognosis, the dataset was divided into high-risk and low-risk groups, based on the median value of the risk score, and the high-risk group had a worse prognosis, compared to the low-risk group, in the TCGA training set (p < 0.001) (Figure 5A). The ROC curve (Figure 5B) was analyzed and the area under the curve (AUC) at 1, 3, and 5 years was found to be 0.756, 0.673, and 0.685, respectively, suggesting that the model is related to the prognosis of the patients, as confirmed by the risk factor plot (Figure 5C). We also validated the model using the GSE14520 dataset, which showed that the high-risk group had a worse prognosis (Figure 5D), with the area under the curve (AUC) being 0.776, 0.590, and 0.623 at 1, 3, and 5 years, respectively (Figure 5E), while the risk factor plots were consistent with those of the training set (Figure 5F). Based on survival analysis, we further analyzed the correlation between the prognostic model and clinical traits, and the results showed that the more advanced the pathological and clinical staging, the higher the prognostic model score (Figure 6A-D).

### Construction of Nomogram plots

**Figure 7:**
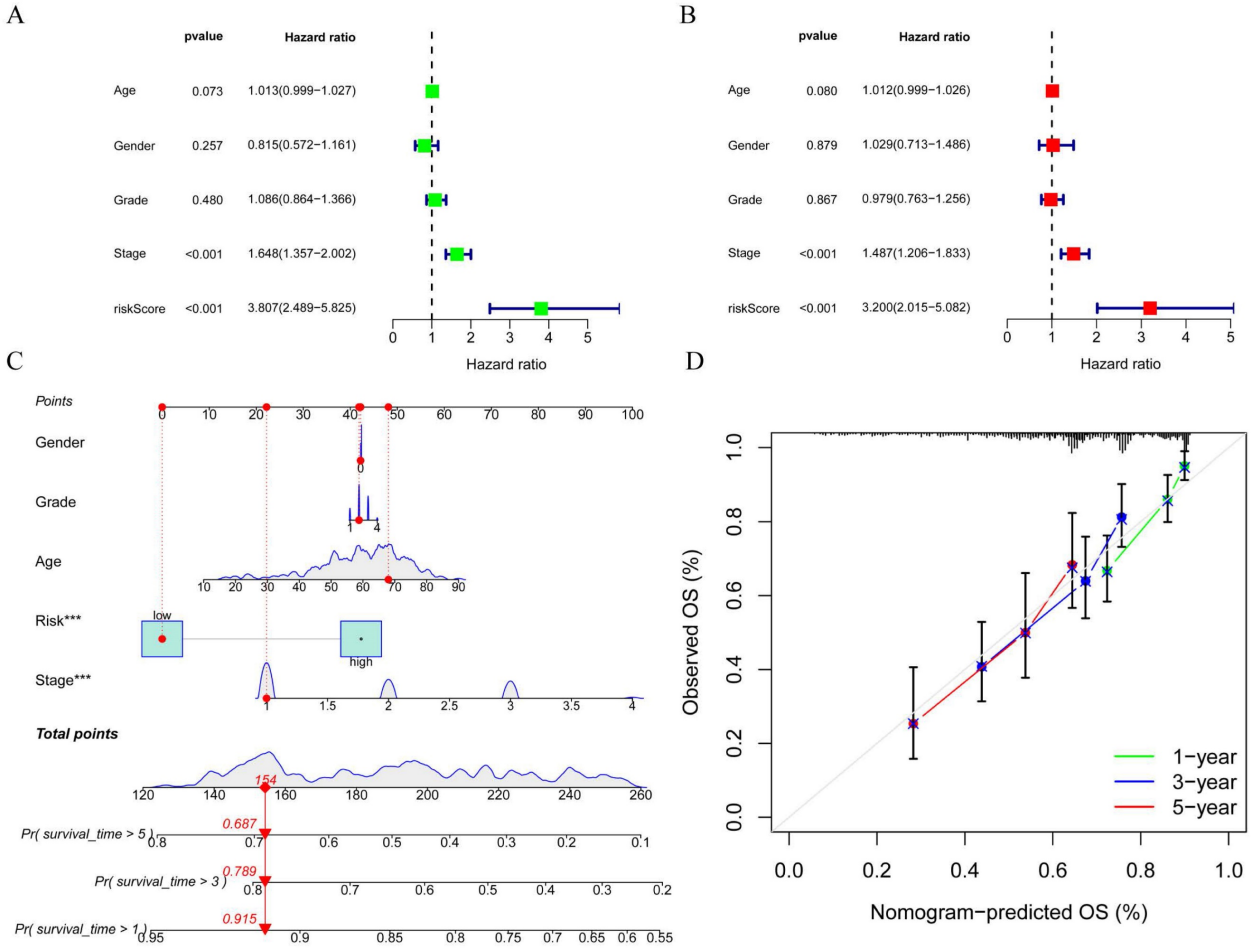
Construction of forest plots and nomograms to better assess the accuracy of the necroptosis model. A. Single-factor COX regression forest plot; B Multi-factor COX regression forest plot; C. Construction of prognostic nomograms; D. Calibration plot.

To better assess the accuracy of the necroptosis model, we performed a univariate Cox analysis by combining clinical data with the Risk score of the prognostic model. To verify whether the risk score we constructed could be used as an independent prognostic factor, we performed a univariate COX regression analysis of the risk score based on clinical traits, and the forest plot showed that the risk score, tumor stage and overall survival were significantly associated, while the risk score had a better prognostic value (HR = 3.807, p < 0.001) (Figure 7A). Multifactorial COX also demonstrated a higher prognostic value for the risk score (HR = 3.200, p < 0.001) (Figure 7B). Based on this, we constructed a Nomogram plot (Figure 7C) and a calibration curve plot (Figure 7D) to assess the predictive effect of the model on the actual result by plotting the fit of the actual probability and the probability predicted by the model under different scenarios in the plot. The results demonstrate that the nomogram is effective in predicting the prognosis of patients, which can be used to guide clinical decisions.

### Enrichment analysis of the differential genes in the high risk and low risk groups and the GSVA analysis

**Figure 8:**
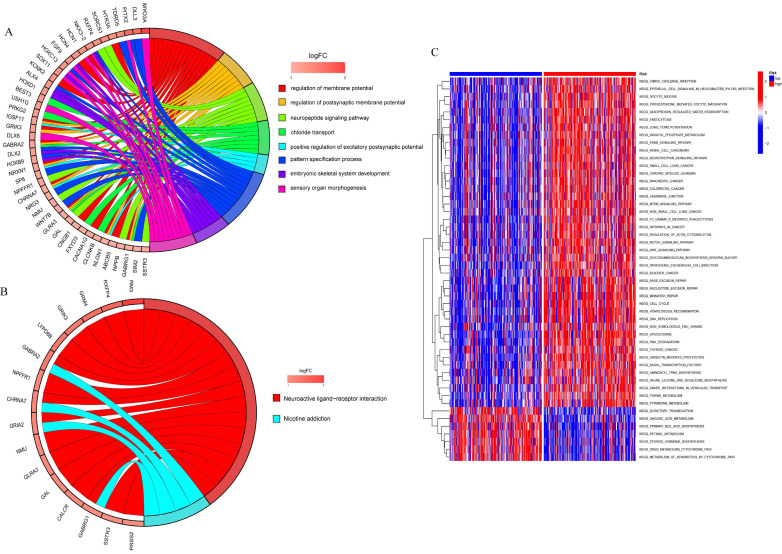
Functional enrichment analysis to analyzed the relationship between differential genes involved in biological processes, molecular functions, cellular components, biological pathways and diseases in the high and low risk groups. A. GO enrichment analysis; B. KEGG enrichment analysis; C. GSVA analysis of high and low risk groups.

To analyze the relationship between differential genes involved in biological processes, molecular functions, cellular components, biological pathways, and diseases in the high- and low-risk groups, we first performed a functional enrichment analysis of differential genes (Figure 8A). The results showed that the genes were mainly enriched in the regulation of the membrane potential, the regulation of the postsynaptic membrane potential, the neuropeptide signaling pathway, chloride transport, the positive regulation of the excitatory postsynaptic potential, the pattern specification process, and the embryonic septum. These functions are related to cellular neurotransmitter transmission and cell and tissue development. KEGG enrichment analysis was performed on differential genes (Figure 8B) and the results were mainly enriched in neuroactive ligand-receptor interaction and nicotine addiction.

GSVA was used to analyze the differential expression of the genes mentioned above between the high-risk and low-risk groups to assess the enrichment of different metabolic pathways between the groups. The results showed that the high-risk group was mainly enriched in DNA replication, mitotic cycle regulation, and various tumor pathways, such as lung, colon, and liver cancers, indicating that prognostic genes are involved in multiple processes that lead to tumorigenesis and growth, and also demonstrate the relevance of prognostic models to liver cancer, which can be used to guide clinical treatment.

### Prognosis-related genes and necroptosis correlation analysis

**Figure 9:**
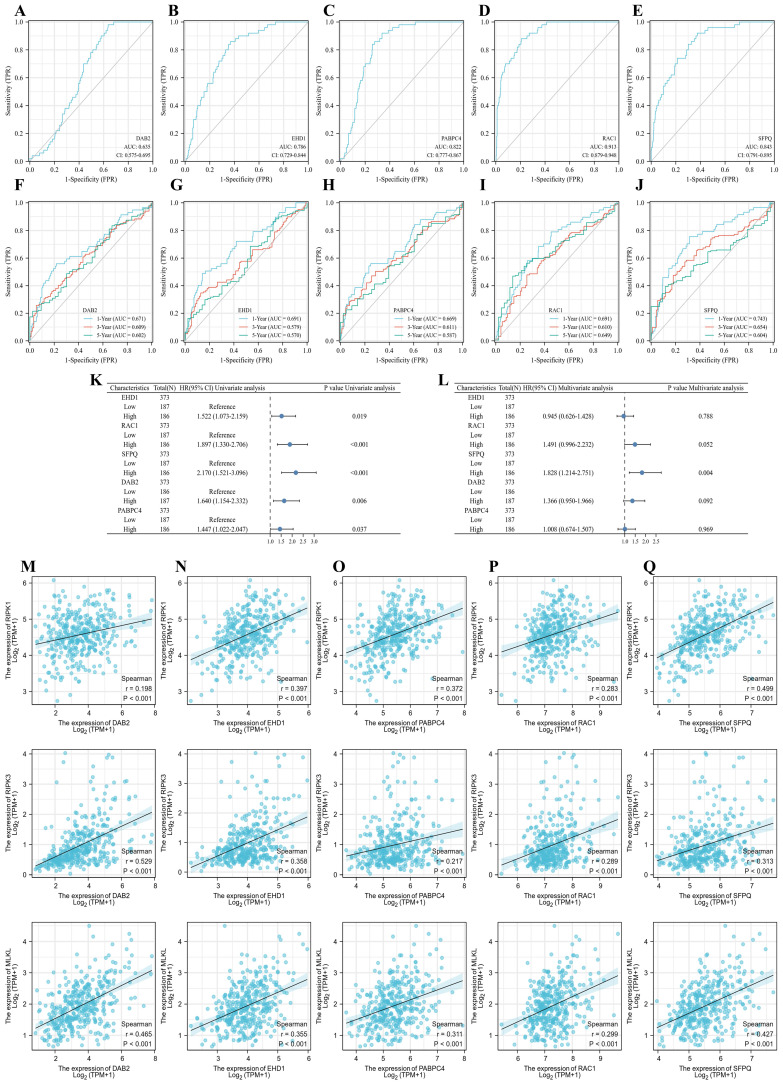
Prognostic genes and necroptosis correlation analysis. A-E. Single gene ROC curve, in order of DAB2, EHD1, PABPC4, RAC1 and SFPQ; F-J. Time dependent ROC curve, in order of DAB2, EHD1, PABPC4, RAC1 and SFPQ; K-L. Single and multifactor Cox regression analysis; M-Q. Scatter plot of prognostic genes DAB2, EHD1, PABPC4, RAC1 and SFPQ correlation with MLKL, RIPK1, RIPK3.

To clarify the effect of the model on tumor prognosis, we also analyzed the effect of single genes on the prognosis of LIHC. eHD1, PABPC4, RAC1, SFPQ and DAB2 were found to be good prognostic indicators, with RAC1 (AUC = 0.913) and SFPQ (AUC = 0.843) being the best (Figure 9A-E). Then we further analyzed the time-dependent AUC curves and the results showed that the SFPQ curve had the best fit (1-year ACU = 0.743,3-year AUC = 0.654 and 5-year AUC = 0.604) (Figure 9F-J). This is consistent with the results of our previous study. Subsequently, we performed a one-way Cox regression analysis and found that all these genes were prognostic influencers. In a subsequent multiway Cox regression analysis, it was found that SFPQ is the main gene that affects prognosis (HR = 1.828, p < 0.004). Next, to verify the correlation between SFPQ and necroptosis, we analyzed the correlation between SFPQ and key pathway proteins: RIPK1, RIPK3, and MLKL. The results showed that SFPQ was positively correlated with RIPK1 (r = 0.499, p < 0.001), RIPK3 (r = 0.313, p < 0.001), and MLKL (r = 0.427, p < 0.001), indicating that SFPQ may be a driver gene that promotes necroptosis.

### Effect of SFPQ on the malignant phenotype and necroptosis of HCC cells

**Figure 10:**
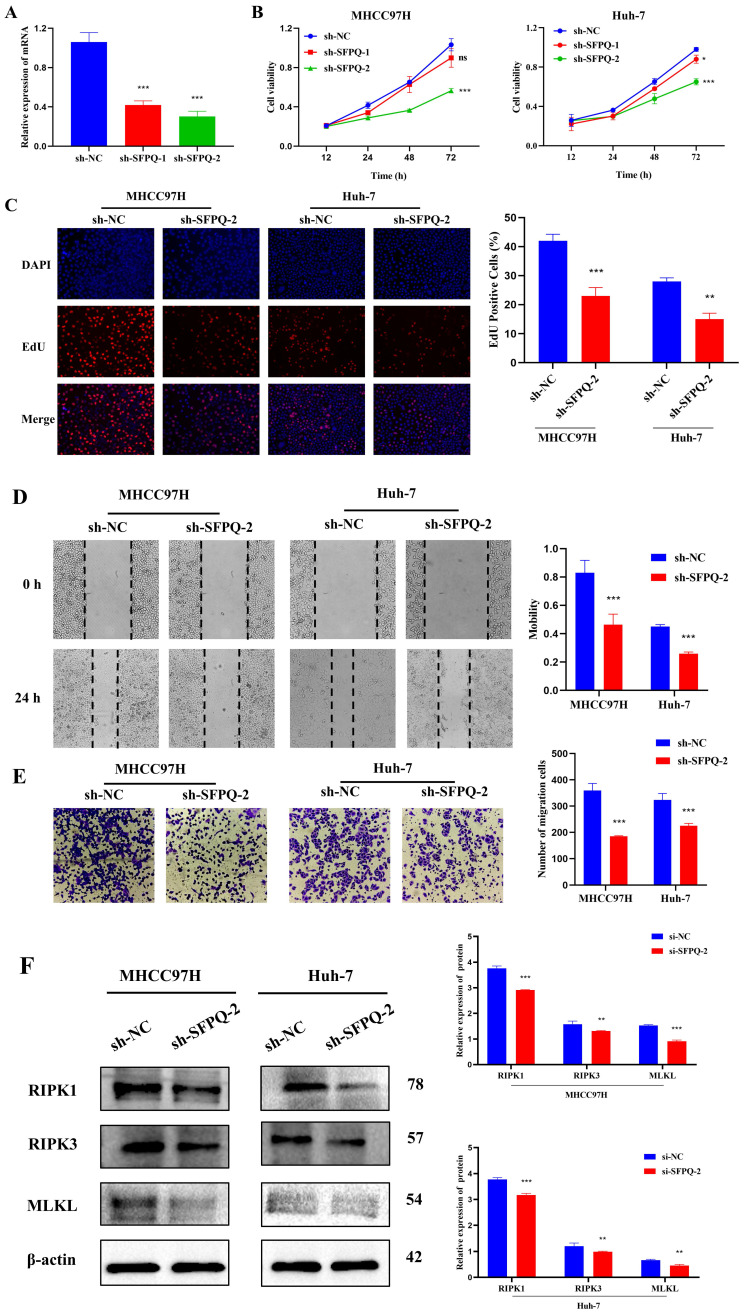
Effect of SFPQ on the malignant phenotype of HCC. A. qRT-PCR assay to detect the expression of SFPQ after shRNA transfection; B-F. Effect of sh-SFPQ transfection on cell proliferation (B and C), would healing (D), migration (E), and on necroptosis-related proteins (F). G. Effect of sh-SFP transfection on cell necrosis. *P < 0.05, **P < 0.01, ***P < 0.001 vs sh-NC.

To further validate the role of SFPQ in HCC, we designed corresponding shRNAs that target the SFPQ gene. qRT PCR analysis showed that the relative expression of sh-SFPQ mRNA was lower than that of the sh-NC group (Figure 10A). The most potent sh-SFPQ-2 was selected for use in subsequent experiments. After SFPQ removal using shRNA, cell viability decreased significantly, compared to the NC group (Figure 10B). To further assess the effect of SFPQ on proliferation, an EdU assay was used. Figure 10C shows that the suppression of SFPQ significantly inhibited the proliferative capacity of the cells. Additionally, we also assessed the effect of SFPQ on migration and invasion. The results of the wound healing assay and the Trans well assay showed that the migration and invasion abilities of the sh-SFPQ-2 group were significantly lower than those of the NC group (Figure 10D-E). The above results indicate that inhibition of SFPQ expression could suppress the malignant phenotype of HCC cells.

In addition, we explore whether there was an association between SFPQ and necroptosis. For validation, we used RIPK1, RIPK3, and MLKL, which are key proteins of necroptosis. The WB results showed that the relative expression of the sh-SFPQ protein was lower than that of the sh-NC group in both groups of HCC cells (Figure 10F). These results further confirm the validity of the prognostic model of necroptosis-related HCC.

## Discussion

HCC is a highly heterogeneous disease with a high incidence and mortality rate that results in a relatively poor prognosis around the world and poses a serious threat to human health and life. Current methods of treatment for HCC include surgery, radiation therapy, interventional radiology, targeted drugs, and immunotherapy, but its prognosis is still not promising^38^. The complexity of the etiology of HCC, whether associated with hepatitis virus infection or metabolic liver disease, leaves many questions unanswered. Therefore, stratified analysis and precision drug use for patients have become the focus of future research on HCC treatment. Along with the advancement of high-throughput genomic technology, a thorough understanding of immune cell subpopulations and microenvironments of different types of cancer, combined with basic and clinical research, has great potential to identify more potential molecular markers.

Necroptosis is a cellular self-destruction process that is activated to prevent blocking apoptosis. Necroptotic cells are usually characterized by rupture of the cell membrane, swelling of organelles, and disintegration of the plasmatic nucleus^39^. Necroptosis is a complementary mode of death due to apoptotic failure, closely associated with inflammation and tumors^40^. Recent studies have reported that liver aging is associated with increased necroptosis, leading to chronic inflammation of the liver, which in turn contributes to the development of liver fibrosis and chronic liver disease^41^. In addition, necroptosis has generated widespread interest in a variety of liver diseases, including HCC, hepatic fibrosis, liver failure, hepatic ischemia-reperfusion injury, and non-alcoholic steatosis^11^, ^42^, ^43^. RIPK3, a central factor of necroptosis, coordinates fatty acid metabolism and hepatocarcinogenesis in tumor-associated macrophages, highlighting a potential strategy that can be used to target immunometabolism in HCC^16^. Similarly, RIPK1 represents an important substrate involved in cell death and inflammation, and studies have shown that RIPK1 and TRAF2 expression in HCC is associated with an unfavorable prognosis^44^. Furthermore, resistance is a major barrier to the use of sorafenib, the first FDA-approved chemotherapy drug for advanced HCC. It was found that sorafenib induces necroptosis in HCC, while HSP90α can block resistance to sorafenib under hypoxic conditions, and in combination with its inhibitor, 17-AAG, is a potential regimen suitable for the treatment of HCC^45^. Therefore, necroptosis-related genes are important for the treatment and prediction of prognosis of malignant tumors. In this study, we constructed a necroptosis prognostic model, exploring and analyzing various databases, which was shown to have a good predictive value for the prognosis of HCC and is expected to guide the treatment of patients with HCC and the early evaluation of the survival prognosis.

In this research study, we performed WGCNA analysis using the GEO and TCGA databases to obtain template genes that are closely associated with the poor prognosis of HCC. The correlation between necroptosis-related genes and the prognosis of HCC was further analyzed and a necroptosis prognostic model was constructed. The prognostic model included five genes, EHD1, RAC1, SFPQ, DAB2, and PABPC4, which are significantly associated with prognosis, which were selected and further analyzed to determine differences in their expression and cellular localization. Finally, we selected the SFPQ gene, which showed the highest prognostic value, for experimental validation. The results demonstrated that inhibition of SFPQ expression could prominently inhibit the viability, proliferative capacity, migration capacity, and invasive capacity of HCC cells. Furthermore, the WB results showed an association between SFPQ and necroptosis. When the expression of SFPQ was inhibited, the relative expression of key necroptosis proteins was also lower than that of the sh-NC group. This result further confirms the validity of the prognostic model of necroptosis-related HCC. To further explore the importance of the necroptosis model as a guide for patient prognosis, we divided the dataset into high-risk and low-risk groups and the results indicated that the high-risk group had a worse prognosis than the low-risk group (p < 0.001), which was confirmed by the ROC curve and risk score. To further evaluate the precision of the model, we constructed a nomogram, and the results indicated that the model was effective in predicting the prognosis of the patient and in guiding clinical decisions. In addition, we further explored differential genes between the high- and low-risk groups using GO and KEGG analyses, and the results showed that the genes were mainly enriched in Neuroactive ligand-receptor interactions and Nicotine addiction. Furthermore, GSVA analysis based on high and low risk groups was used to assess enrichment, and the results showed that the high risk group was mainly enriched in DNA replication, mitotic cycle regulation, and various tumor pathways, such as lung, colon, and liver cancers. Low-risk pathways were mainly enriched in the metabolism of drugs and xenobiotics by cytochrome P450. Together, these results suggest that prognostic genes are involved in multiple processes of tumorigenesis and growth, and further demonstrate that prognostic models are closely associated with liver cancer and can guide clinical treatment.

The KEGG analysis showed that the differential genes were primarily enriched in Neuroactive ligand-receptor interaction and Nicotine addiction, and were most closely associated with the former. All these pathways are mainly involved in cellular neurotransmitter transmission, as well as cell and tissue development. Therefore, we hypothesized that these genes may influence necroptosis between cells by regulating neuroactive ligand-receptor interactions, which accelerate the replication of cancer cells and thus lead to their rapid spread. The neuroactive ligand-receptor interaction signaling pathway is a collection of all receptors and ligands associated with intracellular and extracellular signaling pathways on the plasma membrane. In a study based on the TCGA database, Chen et al. predicted that SYT16 is a prognostic biomarker of low-grade gliomas and was found to be predominantly enriched in the neuroactive ligand-receptor interaction pathway^46^. Similar to our study, Lin et al. used WGCNA and single-cell analysis of differential expression of genes associated with ischemic stroke, which revealed that they were also enriched in the neuroactive ligand-receptor interaction pathway and the calcium signaling pathway^24^. In addition, there are reports on differential genes in lung adenocarcinoma, which indicate that they were also enriched in neuroactive ligand-receptor interactions^47^. Ouyang et al. also confirmed the results of our study in another way. The elevated expression of the Cadherin EGF LAG seven-channel G-type receptor 3 (CELSR3) was shown to be significantly associated with hepatocarcinogenesis and a poor prognosis, and most of its up-regulated genes were enriched in neuroactive ligand-receptor interactions^48^. Furthermore, Zhang et al. used genome-wide DNA to conduct a hydroxymethylation analysis and identified 615 differentially hydroxymethylated regions. Based on the results, related genes were also significantly enriched in neuroactive ligand-receptor interactions^49^. All these previous studies further support the scientific validity and potential clinical significance of this study.

In addition, we summarized the differential expression of the genes mentioned above between the two groups using GSVA and concluded that the high-risk group was mainly enriched in the mTOR signaling pathway, Fc gamma receptor-mediated phagocytosis pathway, the Wnt signaling pathway, cancer pathway, endocytosis, ErbB signaling pathway, renal cell carcinoma, neurotrophin signaling pathways, small cell lung cancer bladder cancer, base excision repair pathway, cell cycle, Spliceosome, thyroid cancer, base transcription factors and SNARE interaction in the vesicular transport, while the low risk group was mainly enriched in the metabolism of drugs and xenobiotics by cytochrome P450. These results suggest that these prognostic genes are involved in multiple processes of tumorigenesis and growth, and also demonstrate that the prognostic model is highly relevant to HCC and can guide clinical treatment. Due to the complexity of the pathogenesis of HCC, cell cycle regulation and signaling as well as multigene interactions at multiple stages are involved. We found that all these pathways are commonly involved in cancer signaling and play an integral role in the development of cancers. mTOR is an immeasurable regulator of cell growth and proliferation, and the signaling pathway that it mediates plays an essential role in the regulation of cell growth and survival. Since much research has been conducted on the mTOR signaling pathway, it was found that it plays a crucial role in the development and progression of diabetes, cancer, and ageing^50^. Xie et al. revealed that the mTOR / RPK3 / necroptosis axis is a driver of intestinal inflammation and cancer. mTOR primarily affects RIPK3 and improves necroptosis induced by TNF and molecular patterns associated with microbial pathogens^51^. As is well known, mTOR is an important downstream protein kinase of the PI3K-Akt signaling pathway and is an influential target for cancer treatment. Shikonin is the main active ingredient in the Chinese medicine "Zicao", which regulates PI3K/AKT/mTOR and MAPK signaling and exerts a strong anticancer effect on various types of cancer by inhibiting RIPK1/3, which ultimately inhibits cell proliferation and induces necroptosis. Studies have suggested that shikonin and its derivatives can be used as potential new drugs for the treatment of cancer and inflammation^52^. Furthermore, in a mouse experiment, the microplastic (MP) and plastic additive, DEHP, induced apoptosis and necroptosis by increasing the expression of RIPK1, RIPK3, and MLKL and decreased the expression of PI3K/AKT/mTOR to activate oxidative stress^53^. The above studies also imply the close association between the mTOR signaling pathway and necroptosis.

The Fc gamma receptor is a class of cell surface receptors that bind to the Fc-terminus of antibodies and whose regulation is highly complex^54^. Park et al. conducted a network-based gene expression analysis, which concluded that the Fcγ receptor-mediated phagocytosis pathway is strongly associated with cognitive function and cerebrospinal fluid biomarkers in Alzheimer's disease^55^. Xin et al. used extensive experiments to demonstrate that ginsenoside Rg3 promotes Fc gamma receptor-mediated phagocytosis through the ERK1/2 and p38 MAPK pathways^56^. Interestingly, GO analysis also indicated that the Lian Hua Qing Wen formula, a herbal compound that exerts its effects against COVID-19, is enriched in Fc gamma receptor-mediated phagocytosis^57^. Despite complex research on the Fc gamma receptor, there are no reports on its relationship with necroptosis, but it is still a promising therapy target in tumors and deserves to be studied in depth for its mechanism of action. On the contrary, the Wnt signaling pathway is a complex and common network in proteins that is most common in embryonic development and cancer. The Wnt signaling pathway is a set of multiple downstream channel signaling pathways that are stimulated by binding of the Wnt ligand protein to membrane protein receptors^58^, ^59^. OSW-1 has previously been shown to be cytotoxic to many types of malignant cells. However, its antitumor mechanism is unclear. Jin et al. found that apoptosis and necroptosis could be induced in HCC cells and the signaling pathway was associated with Wnt, MAPK, VEGF, and P53^60^. Similar to carcinogenesis, the link between the Wnt pathway and necroptosis also extends to hair growth. Zheng et al. investigated Nec-1, a necroptosis inhibitor, which induces the proliferation and migration of outer root sheath cells and increased their hair follicle length in organ cultures from mice and pigs. The mechanism involved inhibition of necroptosis and activation of the Wnt/β-linked protein pathway, which promotes hair growth^61^. Furthermore, the variety of cancer pathways involved suggests that this prognostic model is universal and can exert a good prognostic prediction effect not only in HCC but also in a variety of tumors, such as renal cell carcinoma, small cell lung cancer, bladder cancer, and thyroid cancer, and a large number of studies support this view. Chen et al. predicted that necroptosis-associated genes can act as novel prognostic predictors of the immune microenvironment and treatment response in renal kidney renal clear cell carcinoma^62^. Regarding the study of lung cancer cells, necroptosis is strongly associated with small cell and non-small cell lung cancers^63^, ^64^. In addition, bladder cancer and thyroid cancer, together with necroptosis, have also come under close investigation. In the analysis of necroptosis-related genes with bladder cancer, its potential value in the tumor microenvironment, immunity and prognosis were revealed, providing valuable references for further in-depth investigations into the prognosis of bladder cancer and the development of immunotherapy^65^. Wang et al. further supported this view by using a PKM2 inhibitor to study cisplatin resistance in bladder cancer. Interestingly, they found that PKM2 inhibitors induced necroptosis, and in turn killed cells resistant to cisplatin, and the main cause of this reaction was that cell death was not inhibited by apoptosis inhibitors, but was affected by RIP3 inhibitors or RIP3 siRNA^66^. A similar strong association was also observed in thyroid cancer^67^. Furthermore, the low-risk group was mainly enriched in the metabolism of drugs and xenobiotics by cytochrome P450. Nekvindova et al. showed that cytochrome p450 expression decreased significantly in HCC tissues, compared to normal tissues. Furthermore, patients were more susceptible to drug toxicity and more sensitive to drugs, such as sorafenib^68^. It can be concluded that necroptosis may be involved in the malignant progression of HCC through the pathways mentioned above and that necroptosis-related genes may be prognostic predictors and therapeutic targets for HCC. In summary, in this study, the applicability and scientific validity of the necroptosis prognostic model in cancer was further validated, and a prognostic model for HCC was established to improve the precision of individualized prognosis prediction of patients, which can hopefully provide some guidance for the treatment of patients with HCC.

## Conclusions

HCC is a malignancy with a poor prognosis that can severely affect the health of a population. Along with the gradual development of therapeutic methods, the gaps in this field of research have gradually improved. We downloaded the relevant datasets from TCGA database and then used WGCNA to identify key modules in the necroptosis-related gene set. Single cell datasets were scored using the necroptosis gene set, while differential genes between high and low expression groups were calculated using the WGCNA module genes as the intersection sets to obtain key genes for necroptosis in liver cancer, followed by the construction of prognostic models using LASSO COX regression, followed by multifaceted validation. Finally, the correlation between model genes and key proteins in the necroptosis pathway was calculated to identify the most relevant genes, followed by experimental validation. A prognostic model of necroptosis was constructed in HCC, and five genes, EHD1, RAC1, SFPQ, DAB2, and PABPC4, were found to be significantly associated with the prognosis, while the SFPQ gene was selected to be used for experimental validation as it had the highest prognostic value. The results showed that SFPQ was positively correlated with RIPK1 (r = 0.499, p < 0.001), RIPK3 (r = 0.313, p < 0.001), and MLKL (r = 0.427, p < 0.001), indicating that SFPQ may be a driver gene for the promotion of necroptosis. To explore the implications of the model in guiding patient prognosis, we divided the dataset into high-risk and low-risk groups and performed GO, KEGG, and GSV A analyses on the differential genes between the groups. In addition, the prognostic impact of single genes was further analyzed and the correlation between SFPQ and necroptosis was validated. To further validate the role of SFPQ in HCC, shRNAs that could target the SFPQ gene were designed. The results showed that the prognosis was more unfavorable in the high-risk group, compared to the low-risk group, which was confirmed using the ROC curves and risk factor plots. Furthermore, we further verified the differential genes screened by GO and KEGG analyses and found that they were predominantly enriched in the neuroactive ligand-receptor interaction pathway. The results of the GSVA analysis demonstrated that the high-risk group was mainly enriched in DNA replication, regulation of the mitotic cycle, and regulation of various cancer pathways, while the low-risk group was predominantly enriched in the metabolism of drugs and xenobiotics by cytochrome P450. SFPQ was found to be the main gene affecting the prognosis, and SFPQ was found to be positively correlated with RIPK1, RIPK3, and MLKL. In addition, the knockdown of SFPQ can inhibit the hypermalignant phenotype of HCC cells, and the WB results showed that inhibition of SFPQ expression also resulted in lower expression of necroptosis proteins than in the sh-NC group. All these results also provide novel molecular candidates and interventions that can be used as alternative methods of HCC treatment. However, this study has some limitations. Data were obtained from retrospective samples and there is a lack of clinical trial studies. A larger number of multicenter data are needed to verify our results. Furthermore, the role and function of necroptosis remains to be validated using more advanced methods and techniques. In general, this prognostic model can provide a rapid and accurate assessment of the survival prognosis of patients with HCC, which can serve as a guide for individualized clinical treatment and provide a method of individualized survival prediction and clinical outcome prediction for the use of antitumor immunotherapy in patients with HCC.

## Supplementary Material

Supplementary figures.

## Figures and Tables

**Table 1:** Expression of prognostic genes.

Gene	HR	P value
EHD1	1.795	<0.001
RAC1	1.804	<0.001
SFPQ	2.083	<0.001
DAB2	1.299	<0.001
PABPC4	1.771	<0.001
