# Supplementary material for: Identification and validation of novel signature associated with hepatocellular carcinoma prognosis using Single-cell and WGCNA analysis
Source: Int J Med Sci. 2023 May 11;20(7):870–87. doi: 10.7150/ijms.79274 (PMC10266049; doi:10.7150/ijms.79274)
Supplement: Supplementary file 1 — Supplementary figures. [file ijmsv20p0870s1.pdf]

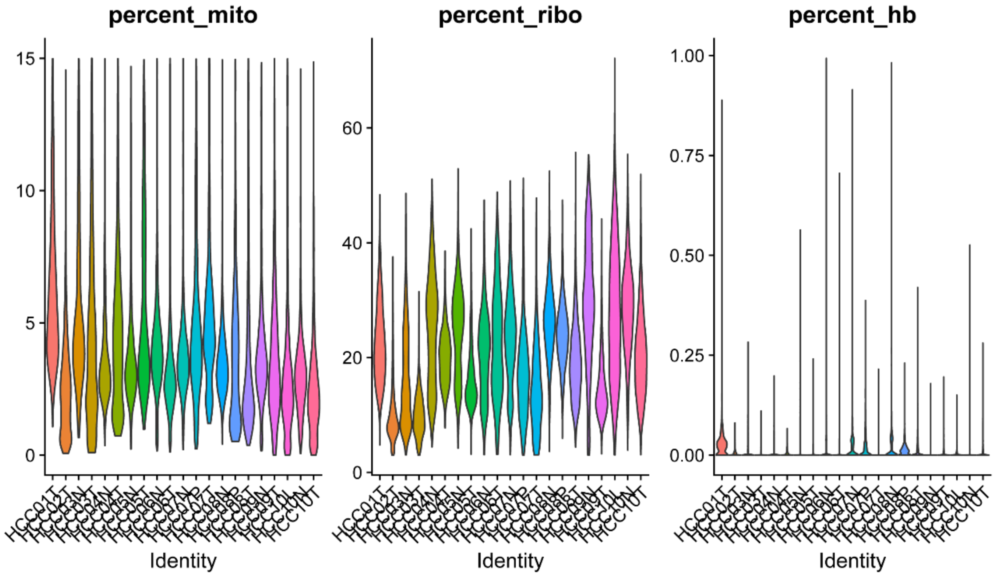

Supplementary figure 1. Mitochondrial, ribosomal and erythrocyte gene ratios for each sample after filtration.

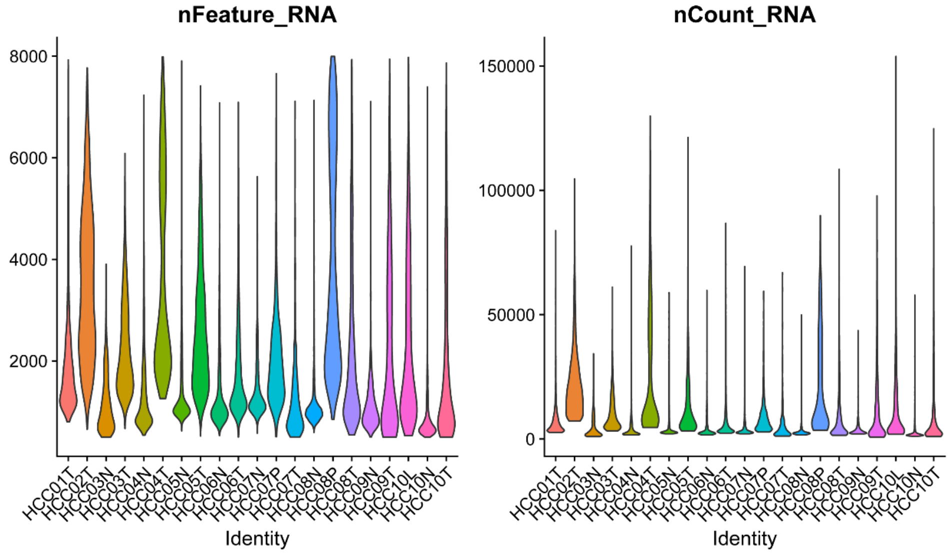

Supplementary figure 2. Mass plot of each sample after single cell filtration.

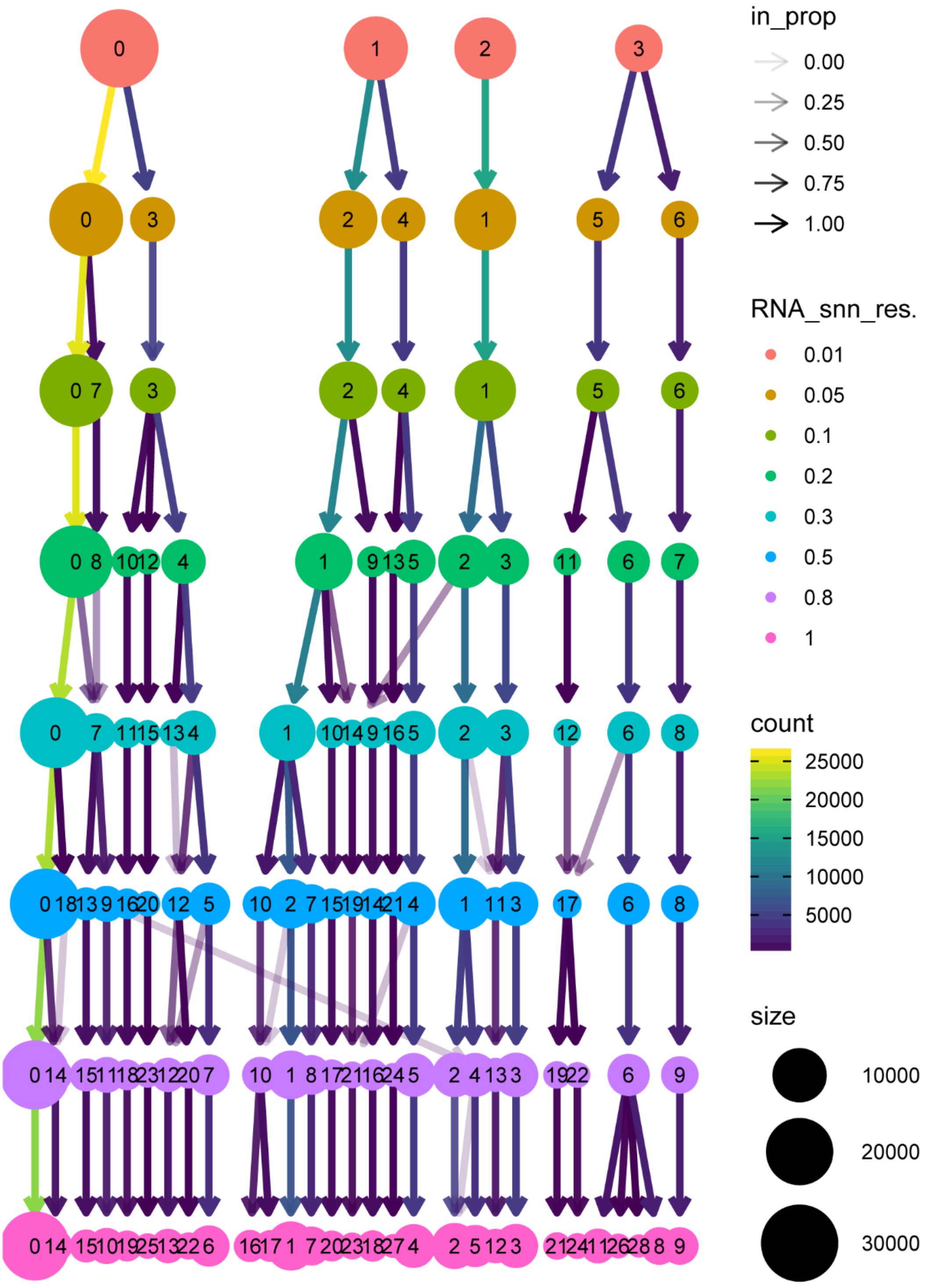

Supplementary figure 3. Resolution in cell clustering trees.
